# The impact of a prior malignancy on outcomes in gastric cancer patients

**DOI:** 10.1002/cam4.3722

**Published:** 2021-01-19

**Authors:** Xiaoyuan Bian, Kaicen Wang, Qiangqiang Wang, Liya Yang, Jiafeng Xia, Wenrui Wu, Lanjuan Li

**Affiliations:** ^1^ State Key Laboratory for Diagnosis and Treatment of Infectious Disease National Clinical Research Center for Infectious Diseases Collaborative Innovation Center for Diagnosis and Treatment of Infectious Diseases The First Affiliated Hospital Zhejiang University School of Medicine Hangzhou P. R. China

**Keywords:** clinical trials, gastric cancer, prior cancer, SEER

## Abstract

**Background:**

The number of cancer survivors has increased rapidly, and there is a higher risk of developing a second cancer. Whether a prior malignancy could affect survival outcomes is unknown. We aimed to investigate the clinical characteristics and prognostic outcomes of prior malignancies in patients with gastric cancer.

**Methods:**

Patient data were extracted from the Surveillance, Epidemiology, and End Results (SEER) database. We used the Kaplan–Meier method, competing risk models, and Cox regression models to evaluate the impact of prior malignancy on survival outcomes.

**Results:**

Among 71,809 patients with primary gastric cancer, 6667 (9.3%) patients had a pre‐existing cancer. Prostate (31.86%), breast (14.34%), and colon and rectum (10.32%) cancer were the most common types. A significant difference was observed in the overall survival rates between patients with and without prior cancer (log‐rank=139.73, *p* < 0.001). In the subgroup analysis, patients with prostate, uterine corpus, lung and bronchus, colon and rectum, esophagus, urinary bladder, leukemia, brain and other nervous system, oral cavity and pharynx, and breast cancer faced inferior survival than those without prior cancer.

**Conclusions:**

A history of prior cancer was associated with worse overall survival in patients with gastric cancer, and the effects varied by different initial cancer types. The exclusion and inclusion of patients who had previous malignancies should be reconsidered according to the specific malignancy types.

## Graphical abstract

In this work, we evaluated the prognosis of gastric cancer among cancer survivors. While much progress had been made in the diagnosis and treatments of primary gastric cancer, little was known regarding clinical significance of subsequent gastric cancer among patients with a history of other malignancies.

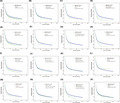

## 1 INTRODUCTION

The number of cancer survivors has increased rapidly and reached approximately fourfold growth with improvements in clinical diagnosis and treatment in the United States.^1^ An increase in overall survival was observed in cancer survivors,^2^ and up to two‐thirds of those survivors could survive for more than 5 years.^1^ There will be approximately 20 million cancer survivors by 2026 due to advanced clinical resources and prolonged life expectations.^3^, ^4^ Therefore, the risk of developing a second malignancy and the treatment of long‐term comorbidities should be taken into consideration.

Gastric cancer is an aggressive and malignant disease with high mortality. According to Cancer Statistics 2020, there will be 27,600 estimated new gastric cancer cases and 11,010 estimated deaths in the United States.^5^ Much progress has been made in prognostic outcomes and therapies for gastric cancer as the first primary malignancy, but the prognosis of gastric cancer as a second cancer remains to be investigated. An increasing number of clinical trials have been conducted to improve the survivorship of patients with gastric cancer. However, individuals with a history of previous malignancy were commonly excluded from patient recruitment.^6^, ^7^, ^8^, ^9^, ^10^ The rationality of the exclusion criterion has hardly been validated and supported by evidence‐based guidelines.^11^ The influence of this exclusion criterion has become a great concern since the number of cancer survivors has grown rapidly. Therefore, it is of great importance to evaluate the enrollment criteria of gastric cancer patients with prior malignancies.

To our knowledge, no study has addressed these issues and determined the exact influence of a previous cancer on the prognosis of patients with gastric cancer. In this study, we aimed to investigate the clinical characteristics and prognostic outcomes of prior malignancies in patients with gastric cancer using the Surveillance, Epidemiology, and End Results (SEER) database.

## 2 METHODS

### 2.1 Study population

Patient data were extracted from the SEER database based on the SEER 18 Registries Custom Data from 1975 to 2016 (November 2018 submission) using SEER*Stat software (version 8.3.6). Approval from our institutional review board was not required due to public access and the use of de‐identified data.

Eligible patients (≥18 years) with primary gastric cancer diagnosed between 2004 and 2015 were recruited from the database. The following patients were excluded from the current study: tumor in situ or benign, inactive follow‐up, unknown survival months and vital status, diagnosed on death autopsy or death certificates only, with more than one prior cancer, or unknown prior cancer information. The included individuals were divided into two groups based on whether they had a previous malignancy. Individuals with prior malignancies were then divided into subgroups based on the prior cancer site, including prostate, uterine corpus, ovary, lung and bronchus, colon and rectum, pancreas, liver and intrahepatic bile duct, esophagus, urinary bladder, kidney and renal pelvis, thyroid, brain and other nervous system, oral cavity and pharynx, breast and other cancer, and melanoma of the skin and leukemia. To avoid the impact of synchronous malignancy, patients with a prior cancer diagnosed within 6 months or with prior gastric cancer were excluded.

### 2.2 Covariates and outcomes

Demographic characteristics included age at diagnosis (≤70 years and >70 years), sex (male and female), race (white, black, American Indian and Alaska Native, Asian/Pacific Islander, and unknown), marital status (married, unmarried, and unknown), and insurance (yes, no, and unknown). Tumor and therapy factors included tumor site (cardia, body/fundus, antrum/pylorus, and others), tumor size (≤ 1 cm, 1–3 cm, 3–5 cm, >5 cm, and unknown), histological type (adenocarcinoma, mucinous cell adenocarcinoma, signet ring cell carcinoma, and others), 6th American Joint Committee on Cancer (AJCC) stage (I, II, III, IV, and others), grade (well differentiated, moderately differentiated, poorly differentiated, undifferentiated, and unknown), surgery (yes, no, and unknown), radiation (yes, no, and unknown), and chemotherapy (yes, no/unknown). In addition, the stage and interval time of prior cancer were obtained from the database.

Information on outcomes, including survival months, vital status (alive and dead) and cause of death (due to gastric cancer, due to other causes, and unknown) were also abstracted. Overall survival was defined as the survival time from the diagnosis of gastric cancer to any cause of death, and gastric cancer‐specific survival was defined as the time from gastric cancer diagnosis to death from gastric cancer.

### 2.3 Statistical analysis

The descriptive statistics of patients’ demographic, tumor, and therapy characteristics were analyzed using the Chi‐square test. The site and stage distribution of the previous malignancies were calculated and presented. Additionally, the median interval time from prior cancer diagnosis to subsequent gastric cancer diagnosis was also calculated. Kaplan–Meier curves and the log‐rank test were performed to compare the overall survival between patients with and without prior cancer. A competing risk model was used to compare gastric cancer‐specific mortality and non‐gastric cancer‐specific mortality. Furthermore, we formulated nomograms based on multivariate survival analysis. The discrimination and calibration of the nomograms were evaluated by the C‐index and calibration plots. Hazard ratios (HRs) of overall and gastric cancer‐specific survival were estimated using the multivariable Cox model after adjusting for the covariates of interest (prior cancer, age at diagnosis, sex, race, insurance, marital status, tumor site, histological type, tumor size, 6th AJCC stage, grade, surgery, radiation, and chemotherapy). Subgroup survival analyses were performed to determine whether prognostic outcomes were differentially associated with prior malignancy types. SPSS (version 20.0), R software (version 3.6.2), and GraphPad Prism (version 7.0) were used to conduct the statistical analyses and generate images. A significant difference was defined as a two‐tailed *p* value <0.05.

## 3 RESULTS

In our study, 71,809 primary gastric cancer patients were identified on the basis of the inclusion and exclusion criteria. Among the included cohort, 6667 (9.3%) patients had a history of previous cancer, while 65,142 (90.7%) patients did not (Table 1).

**TABLE 1:** Baseline patient characteristics of the gastric cancer

Patient characteristics	Total number N = 71,809	With prior cancer N = 6667 (9.3%)		No prior cancer N = 65,142 (90.7%)		*p*
Age (median)		75 years		78 years		
Sex						<0.001
Male		4433	(66.5%)	39,289	(60.3%)	
Female		2234	(33.5%)	25,853	(39.7%)	
Tumor Site						<0.001
Cardia		2095	(31.4%)	18,512	(28.4%)	
Body/fundus		975	(14.6%)	9724	(14.9%)	
Antrum/pylorus		1310	(19.6%)	13,394	(20.6%)	
Others		2287	(34.3%)	23,512	(36.1%)	
Histological Type						<0.001
Adeno		4826	(72.4%)	45,419	(69.7%)	
Mucinous		128	(1.9%)	1222	(1.9%)	
Signet ring cell		1007	(15.1%)	11,325	(17.4%)	
Others		706	(10.6%)	7176	(11.0%)	
Tumor Size						<0.001
≤1 cm		381	(5.7%)	3575	(5.5%)	
1–3 cm		1055	(15.8%)	9743	(15.0%)	
3–5 cm		1052	(15.8%)	10,020	(15.4%)	
>5 cm		1320	(19.8%)	15,080	(23.1%)	
Unknown		2859	(42.9%)	26,724	(41.0%)	
AJCC stage (6th)						<0.001
I		1749	(26.2%)	14,254	(21.9%)	
II		668	(10.0%)	6603	(10.1%)	
III		547	(8.2%)	6171	(9.5%)	
IV		2099	(31.5%)	23,793	(36.5%)	
Others		1604	(24.1%)	14,321	(22.0%)	
Grade						0.002
Well		404	(6.1%)	3971	(6.1%)	
Moderately		1491	(22.4%)	13,274	(20.4%)	
Poorly		3195	(47.9%)	32,267	(49.5%)	
Undifferentiated		124	(1.9%)	1401	(2.2%)	
Unknown		1453	(21.8%)	14,229	(21.8%)	
Surgery						<0.001
Yes		2857	(42.9%)	30,800	(47.3%)	
No		3720	(55.8%)	33,411	(51.3%)	
Unknown		90	(1.3%)	931	(1.4%)	
Radiation						<0.001
Yes		631	(9.5%)	8423	(12.9%)	
No		6032	(90.5%)	56,687	(87.0%)	
Unknown		4	(0.1%)	32	(0.0%)	
Chemotherapy						<0.001
Yes		2455	(36.8%)	28,873	(44.3%)	
No/Unknown		4212	(63.2%)	36,269	(55.7%)	
Race						<0.001
White		4956	(74.3%)	45,825	(70.3%)	
Black		904	(13.6%)	9015	(13.8%)	
AI/AN		47	(0.7%)	588	(0.9%)	
AP		759	(11.4%)	9346	(14.3%)	
Unknown		1	(0.0%)	368	(0.6%)	
Insurance						<0.001
Yes		5022	(75.3%)	45,544	(69.9%)	
No		57	(0.9%)	1989	(3.1%)	
Unknown		1588	(23.8%)	17,609	(27.0%)	
Marital status						<0.001
Married		3849	(57.7%)	35,879	(55.1%)	
Unmarried		2391	(35.9%)	25,600	(39.3%)	
Unknown		427	(6.4%)	3663	(5.6%)	

Abbreviations: Adeno, Adenocarcinoma; AI/AN, American Indian/Alaska Native; AP, Asian or Pacific Islander; Mucinous, Mucinous cell adenocarcinoma; Signet ring cell, signet ring cell carcinoma.

### 3.1 Demographics and clinical characteristics of the cohort

There were a number of differences in clinicopathological characteristics between patients with and without pre‐existing malignancies. As presented in Table 1, a higher proportion of individuals with a prior malignancy were younger, white, and married. Compared to patients without prior cancer, those with a history of prior cancer had smaller tumor sizes (19.8% vs. 23.1%, *p* < 0.001), fewer of the signet ring cell histological type (15.1% vs. 17.4%, *p* < 0.001), and less advanced stages (26.2% vs. 21.9%, *p* < 0.001) and grades (47.9% vs. 49.5%, *p* = 0.002). For the therapy factors, individuals with a prior cancer were less likely to undergo surgery (42.9% vs. 47.3%, *p* < 0.001), radiation (9.5% vs. 12.9%, *p* < 0.001), and chemotherapy (36.8% vs. 44.3%, *p* < 0.001) and had more insurance protection (75.3% vs. 69.9%, *p* < 0.001).

**FIGURE 1:**
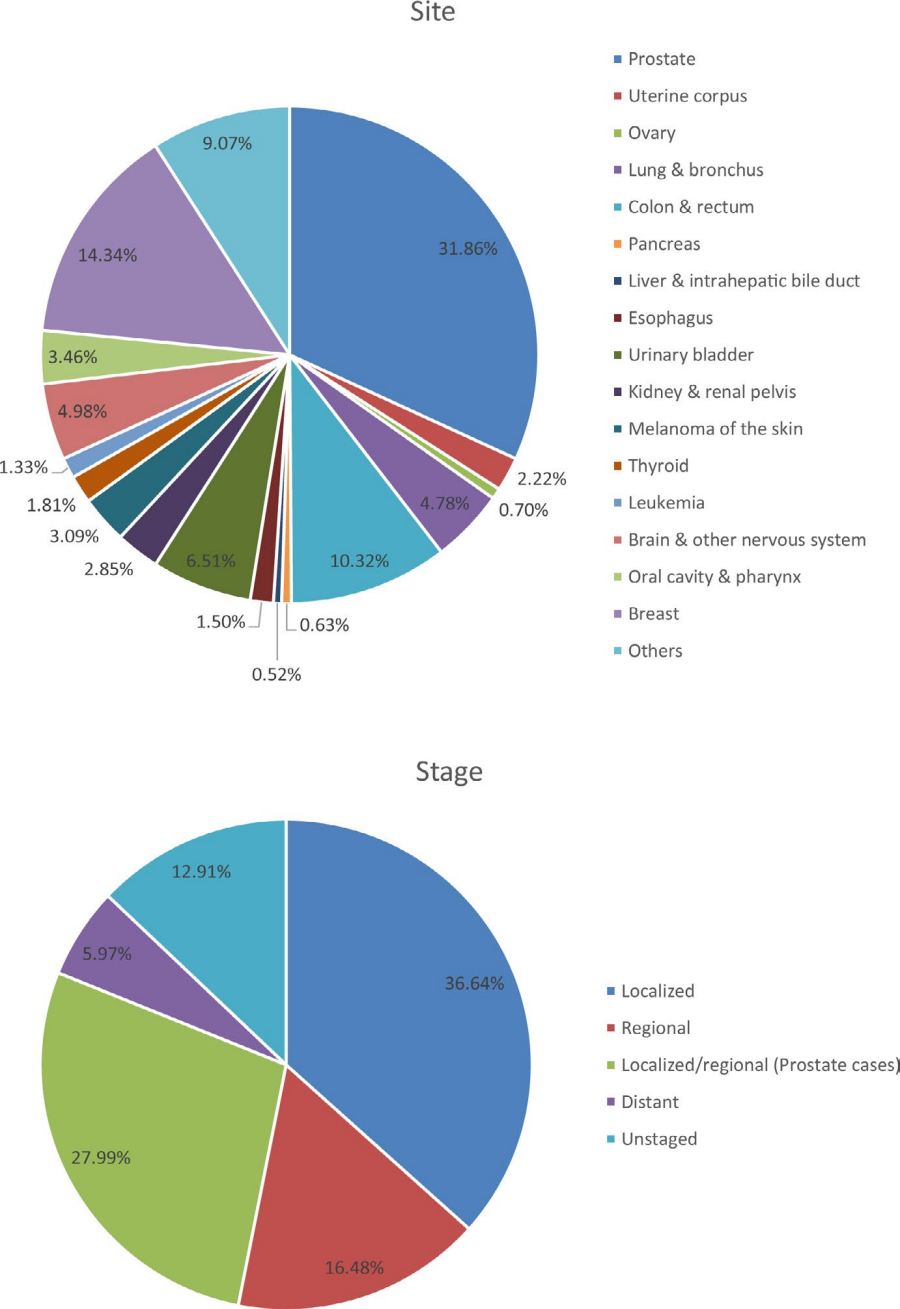
Site (upper panel) and stage (down panel) distributions of prior cancer for patients with gastric cancer

**FIGURE 2:**
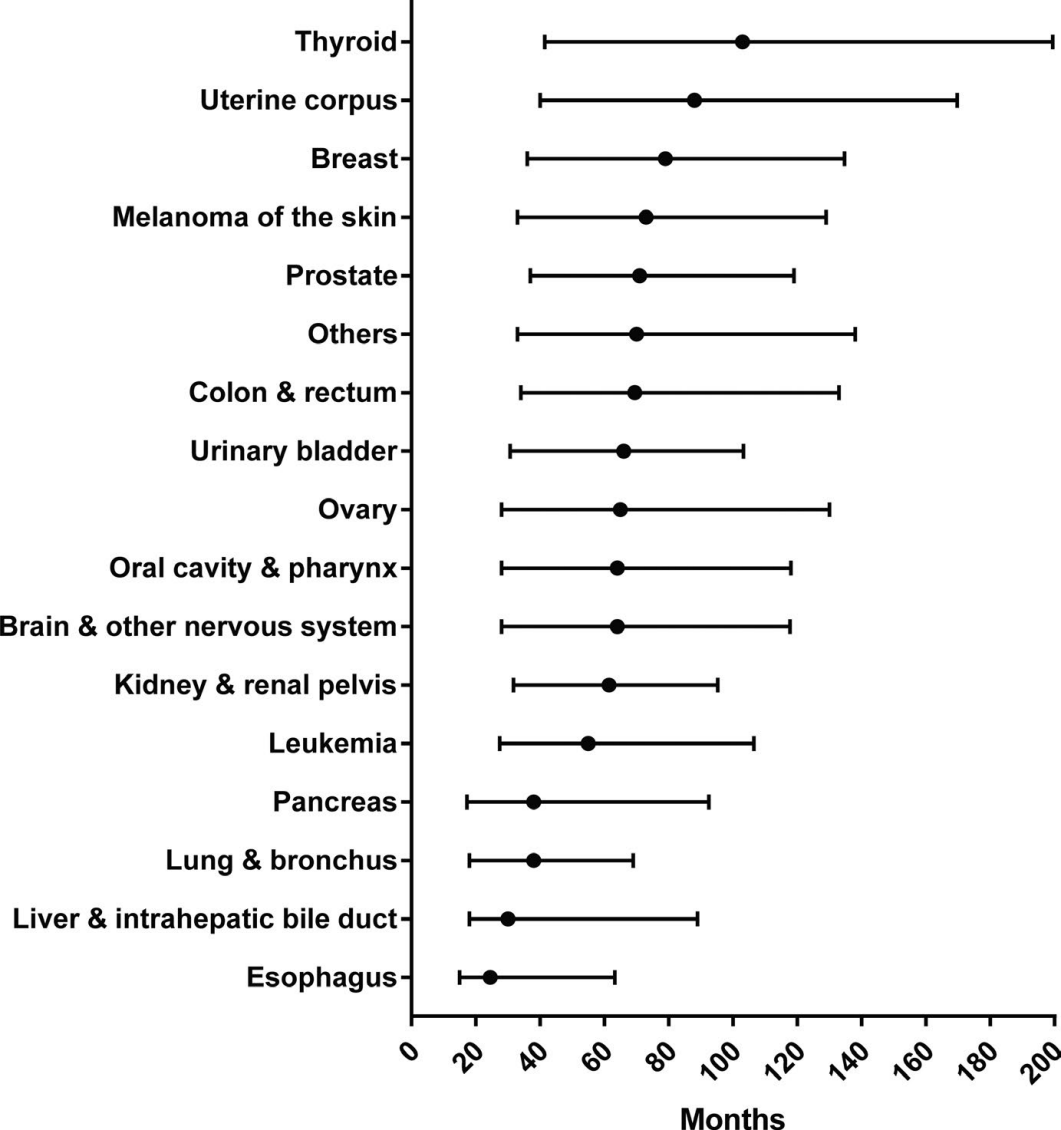
The median interval time between prior cancer and gastric cancer stratified by prior cancer sites

Among the different prior cancer types, prostate (31.86%), breast (14.34%), and colon and rectum (10.32%) were the most common sites (Figure 1). Regarding the prior cancer stage distribution, the majority of patients with a previous malignancy were at a localized or regional stage (81.12%), while only 5.97% patients were at a distant stage (Figure 1). The median period of time from prior malignancy diagnosis to the subsequent gastric cancer diagnosis was 68 months. For most cancer survivors, the median time interval was more than 60 months, while the median time of survivors with esophagus (24.5 months), liver and intrahepatic bile duct (30 months), pancreatic (38 months), and lung and bronchus (38 months) cancer was less than 40 months (Figure 2).

### 3.2 Comparison of survival rates and mortality

**FIGURE 3:**
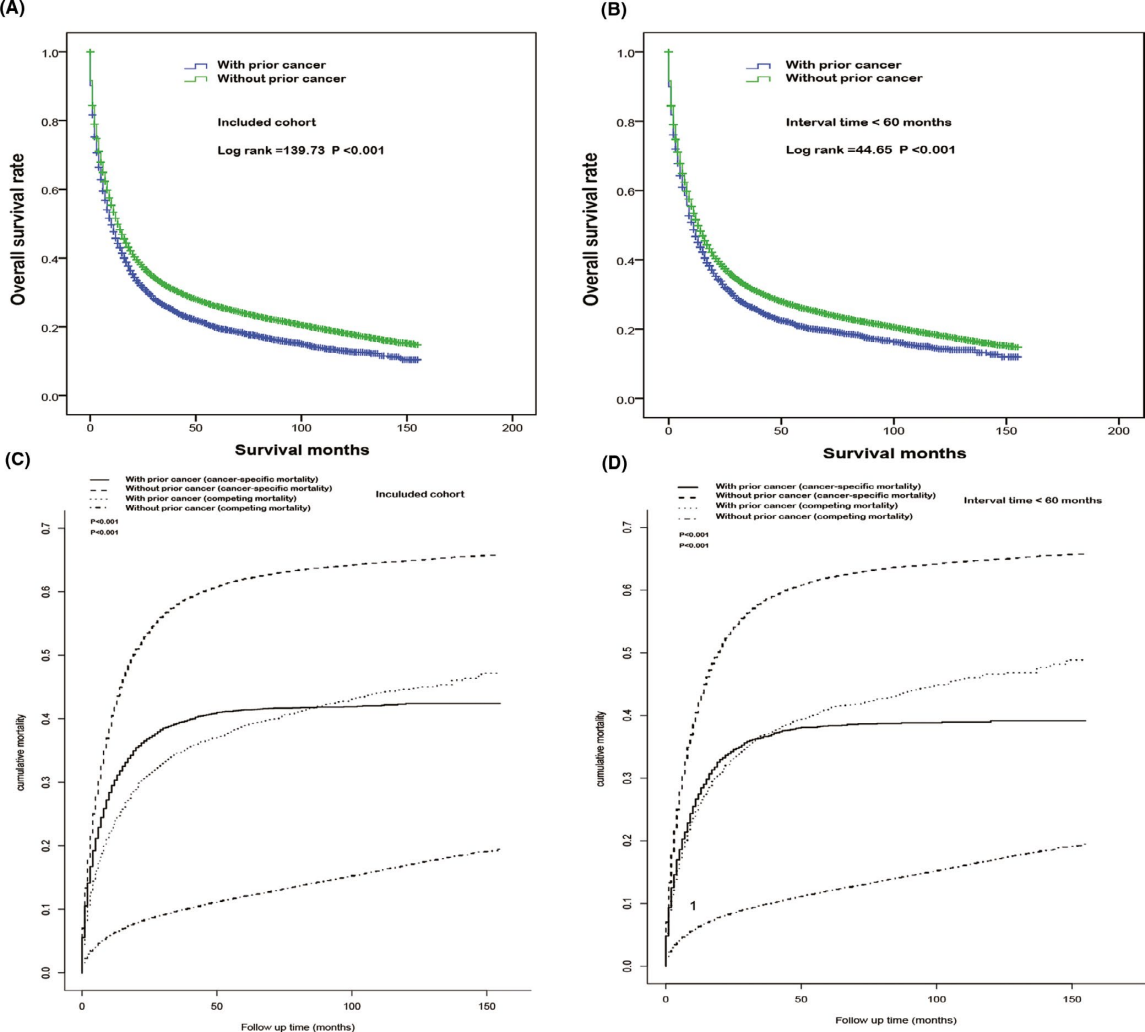
Overall survival analysis of patients with gastric cancer (A) and patients with a prior cancer diagnosed within 60 months (B). Competing analysis of patients with gastric cancer (C) and patients with a prior cancer diagnosed within 60 months (D)

A significant difference was observed in the overall survival rates between patients with and without prior cancer (log‐rank=139.73, *p* < 0.001, Figure 3A). As far as we know, 60‐month exclusion is commonly used in clinical trials.^12^ Similarly, we found a marked difference in survival rates between patients without prior cancer and those with prior cancer within 60 months (log‐rank=44.65, *p* < 0.001, Figure 3B). Cancer‐specific mortality was considered a competing event compared to non‐cancer‐specific mortality. Therefore, we adopted the competing risk model to compare the gastric and non‐gastric cancer‐specific mortalities between patients with and without a pre‐existing cancer. As shown in Figure 3C, patients with a previous malignancy significantly differed from those without previous malignancy in both gastric and non‐gastric cancer‐specific mortality (both *p* < 0.001). Similar results were also found in the cohort with interval times less than 60 months (both *p* < 0.001, Figure 3D).

**FIGURE 4:**
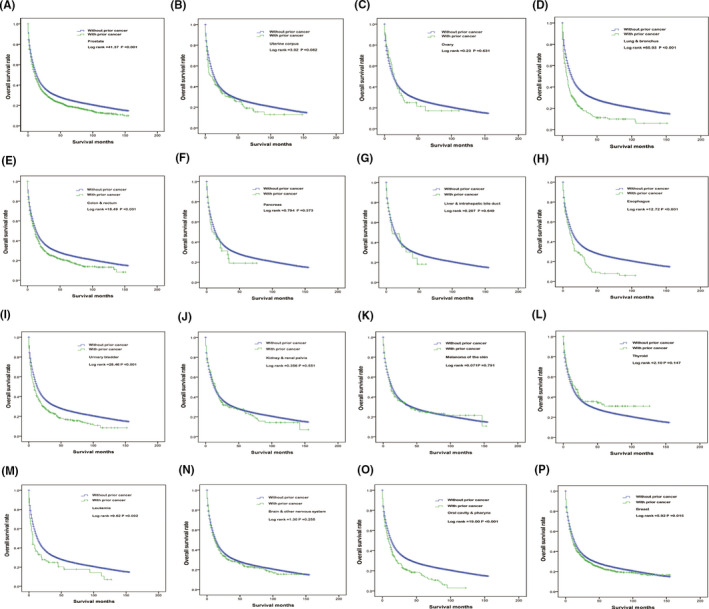
Overall survival analysis of patients with gastric cancer who had a prior cancer: (A) prostate; (B) uterine corpus; (C) ovary; (D) lung and bronchus; (E) colon and rectum; (F) pancreas; (G) liver and intrahepatic bile duct; (H) esophagus; (I) urinary bladder; (J) kidney and renal pelvis; (K) melanoma of the skin; (L) thyroid; (M) leukemia; (N) brain and other nervous system; (O) oral cavity and pharynx. (P) breast

As Table 2 shows, the 3‐year and 5‐year overall survival rates of patients with prior cancer were 45.8% and 25.9%, respectively, while those without a previous cancer were 51.6% and 31.9%. Among the cancer survivors, those with thyroid (55.6%), kidney and renal pelvis (54.1%), and ovarian (53.2%) cancer had better 3‐year survival rates, and those with lung and bronchus cancer had the worst 3‐year and 5‐year survival rates (32.8% and 16.0%, respectively, Table 2 and Figure 4).

**TABLE 2:** 3‐year and 5‐year overall survival rate of gastric cancer patients stratified by prior cancer site

Previous cancer site	Overall survival rate (%)	
	3‐year (95%CI)	5‐year (95%CI)
No prior cancer	51.6 (51.4, 51.8)	31.9 (31.7, 32.1)
With prior cancer	45.8 (45.2, 46.4)	25.9 (25.3, 26.5)
Prostate	46.6 (45.5, 47.7)	26.5 (25.5, 27.5)
Uterine corpus	46.4 (42.3, 50.5)	29.6 (25.7, 33.5)
Ovary	53.2 (45.9, 60.5)	25.1 (18.7, 31.5)
Lung and bronchus	32.8 (30.2, 35.4)	16.0 (13.8, 18.2)
Colon and rectum	46.5 (44.6, 48.4)	25.0 (23.3, 26.7)
Pancreas	47.6 (39.9, 55.3)	19.0 (11.8, 26.2)
Liver and intrahepatic bile duct	48.6 (40.2, 57.0)	20.4 (17.4, 23.4)
Esophagus	44.0 (39.0, 49.0)	18.6 (14.6, 22.6)
Urinary bladder	41.2 (38.8, 43.6)	23.5 (21.4, 25.6)
Kidney and renal pelvis	54.1 (50.5, 57.7)	29.6 (26.1,33.1)
Melanoma of the skin	47.1 (43.6, 50.6)	30.9 (27.6, 34.2)
Thyroid	55.6 (51.0, 60.2)	34.4 (29.8, 39.0)
Leukemia	36.7 (31.6, 41.8)	24.7 (19.9, 29.5)
Brain and other nervous system	49.4 (46.6, 52.2)	28.7 (26.1, 31.3)
Oral cavity and pharynx	43.5 (40.2, 46.8)	20.4 (17.6, 23.2)
Breast	47.2 (45.6, 48.8)	29.0 (27.5, 30.5)

### 3.3 Multivariable analyses of overall and cancer‐specific survival

**FIGURE 5:**
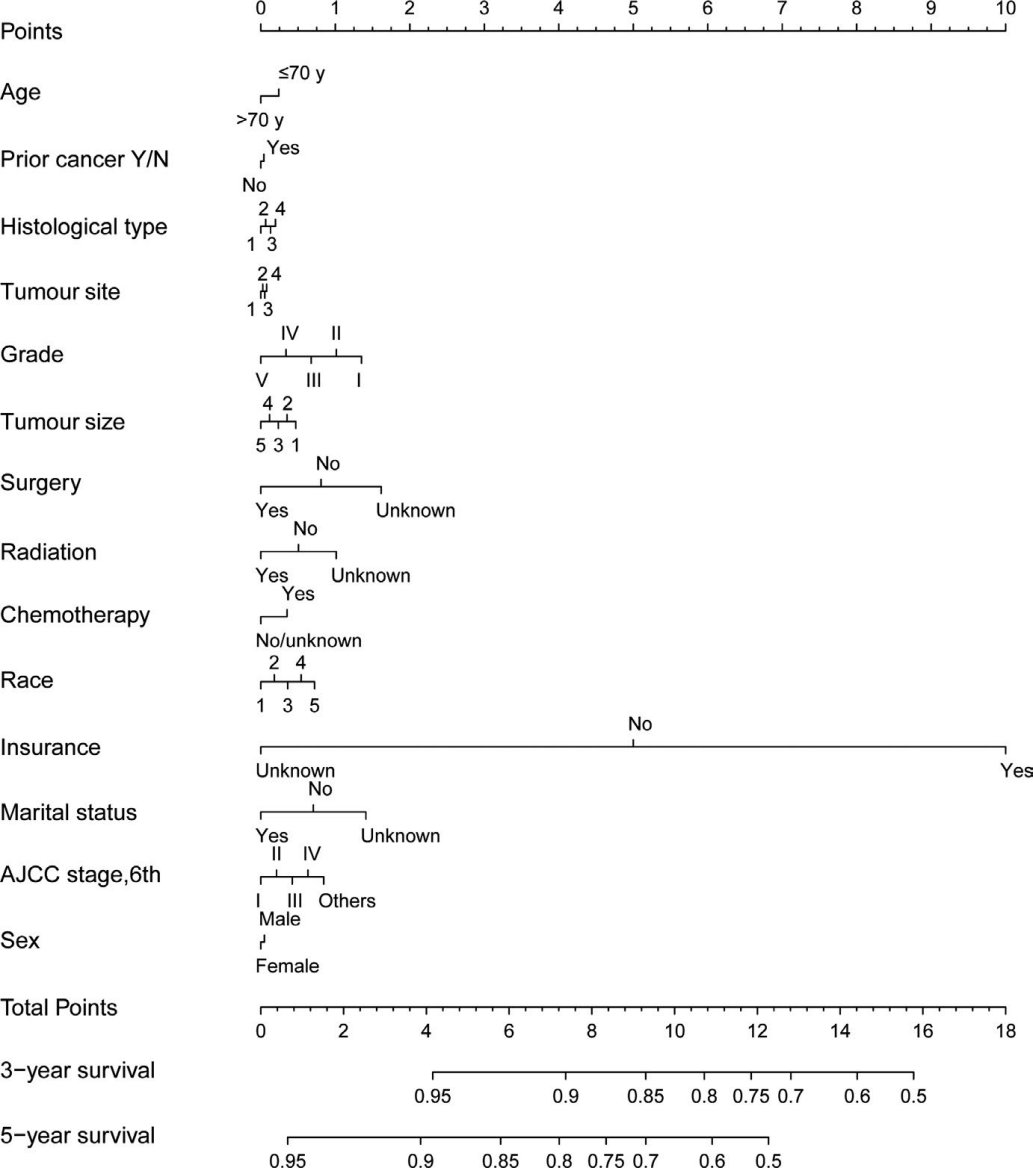
The nomogram for predicting overall survival in gastric cancer patients with and without a prior cancer. Abbreviations: Histologic type, 1, Adenocarcinoma; 2, Mucinous cell adenocarcinoma; 3, Signet ring cell. Tumor site, 1, cardia; 2, body/fundus; 3, antrum/pylorus; 4, others. Tumor size, 1, ≤1 cm; 2, 1–3 cm; 3, 3–5 cm; 4, >5 cm; 5, unknown. Race, 1, white; 2, black; 3, American Indian/Alaska Native; 4, Asian or Pacific Islander; 5, unknown. AJCC, American Joint Committee on Cancer

**FIGURE 6:**
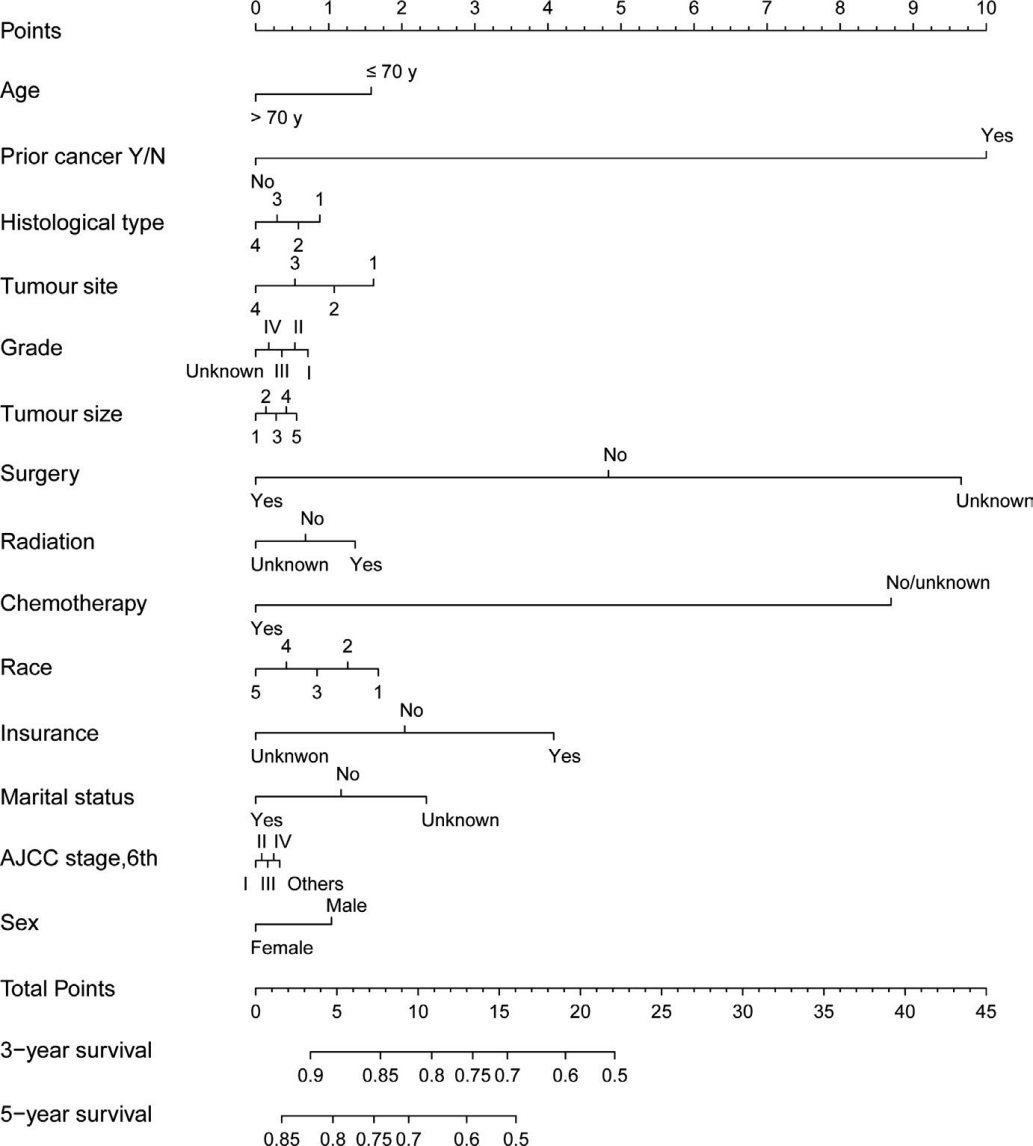
The nomogram for predicting gastric cancer‐specific survival in gastric cancer patients with and without a prior cancer. Abbreviations: Histologic type, 1, Adenocarcinoma; 2, Mucinous cell adenocarcinoma; 3, Signet ring cell. Tumor site, 1, cardia; 2, body/fundus; 3, antrum/pylorus; 4, others. Tumor size, 1, ≤1 cm; 2, 1–3 cm; 3, 3–5 cm; 4, >5 cm; 5, unknown. Race, 1, white; 2, black; 3, American Indian/Alaska Native; 4, Asian or Pacific Islander; 5, unknown. AJCC, American Joint Committee on Cancer

Next, we constructed the nomogram to explore the multivariable prognostic factors of patients with gastric cancer (Figures 5 and 6). The discriminative abilities of the overall and gastric cancer‐specific predictions were evaluated by calculating the C‐indexes, which were 0.624 and 0.720, respectively. Additionally, the 3‐year and 5‐year calibration curves are presented in Figures S1 and S2.

We further adopted a multivariate Cox regression model to evaluate all independent factors for overall and gastric cancer‐specific prognostic outcomes (Table 3). A history of cancer was associated with worse overall and better gastric cancer‐specific survival after adjusting for other covariates (HR=0.79, 95% CI=0.76–0.82, *p* < 0.001; and HR=1.24, 95% CI=1.18–1.30, *p* < 0.001, respectively). In the subgroup analysis, patients with prostate, lung and bronchus, colon and rectum, esophagus, urinary bladder, brain and other nervous system, oral cavity and pharynx, and breast cancer faced inferior overall survival and better cancer‐specific survival than those without prior cancer (Table 4). Interestingly, patients with uterine corpus cancer and leukemia had worse overall survival but similar gastric cancer‐specific survival, while patients with ovarian, liver and intrahepatic bile duct, kidney and renal pelvis, and melanoma of the skin cancer had better gastric cancer‐specific survival but similar overall survival. In addition, patients with thyroid cancer were not associated with worse survival (Table 4).

**TABLE 3:** Multivariable Cox regression analysis of overall and gastric cancer‐specific survival in patients with gastric cancer

Characteristics	Overall adjusted HR	*p*	Gastric cancer‐specific adjusted HR	*p*
Prior cancer				
Yes	Reference		Reference	
No	0.79 (0.76, 0.82)	<0.001	1.24 (1.18, 1.30)	<0.001
Age at diagnoses				
≤ 70 years	Reference		Reference	
> 70 years	1.33 (1.25, 1.41)	<0.001	1.42 (1.31, 1.54)	<0.001
Sex		<0.001		<0.001
Male	Reference		Reference	
Female	0.89 (0.87, 0.90)	<0.001	0.91 (0.89, 0.93)	<0.001
Tumor Site		<0.001		<0.001
Cardia	Reference		Reference	
Body/fundus	0.90 (0.88, 0.93)	<0.001	0.94 (0.91, 0.98)	<0.001
Antrum/pylorus	1.00 (0.97, 1.03)	0.880	1.04 (1.01, 1.07)	0.009
Others	1.01 (0.99, 1.04)	0.315	1.07 (1.04, 1.09)	<0.001
Histological Type		<0.001		<0.001
Adeno	Reference		Reference	
Mucinous	1.10 (1.04, 1.17)	0.002	1.13 (1.06, 1.21)	<0.001
Signet ring cell	1.11 (1.09, 1.14)	<0.001	1.14 (1.12, 1.17)	<0.001
Others	0.83 (0.80, 0.86)	<0.001	0.82 (0.79, 0.86)	<0.001
Tumor Size		<0.001		<0.001
≤ 1 cm	Reference		Reference	
1–3 cm	1.55 (1.46, 1.64)	<0.001	1.95 (1.80, 2.11)	<0.001
3–5 cm	1.95 (1.84, 2.07)	<0.001	2.52 (2.33, 2.73)	<0.001
>5 cm	2.08 (1.96, 2.20)	<0.001	2.77 (2.56, 2.99)	<0.001
Unknown	2.07 (1.95, 2.18)	<0.001	2.70 (2.50, 2.92)	<0.001
AJCC stage (6th)		<0.001		<0.001
I	Reference		Reference	
II	1.60 (1.54, 1.66)	<0.001	1.93 (1.85, 2.01)	<0.001
III	2.14 (2.07, 2.22)	<0.001	2.72 (2.61, 2.84)	<0.001
IV	2.80 (2.72, 2.88)	<0.001	3.61 (3.50, 3.74)	<0.001
Others	1.00 (0.97, 1.03)	0.846	1.09 (1.04, 1.13)	<0.001
Grade		<0.001		<0.001
Well	Reference		Reference	
Moderately	1.78 (1.69, 1.88)	<0.001	2.09 (1.95, 2.34)	<0.001
Poorly	2.20 (2.09, 2.31)	<0.001	2.73 (2.55, 2.91)	<0.001
Undifferentiated	2.21 (2.05, 2.39)	<0.001	2.80 (2.55, 3.07)	<0.001
Unknown	1.51 (1.43, 1.59)	<0.001	1.80 (1.69, 1.93)	<0.001
Surgery		<0.001		<0.001
Yes	Reference		Reference	
No	3.23 (3.15, 3.31)	<0.001	3.44 (3.34, 3.54)	<0.001
Unknown	2.23 (2.06, 2.41)	<0.001	2.49 (2.29, 2.71)	<0.001
Radiation		<0.001		<0.001
Yes	Reference		Reference	
No	0.92 (0.89, 0.95)	<0.001	0.89 (0.86, 0.93)	<0.001
Unknown	1.02 (0.69, 1.49)	0.940	1.13 (0.75,1.69)	0.563
Chemotherapy		<0.001		<0.001
Yes	Reference		Reference	
No/Unknown	2.06 (2.02, 2.10)	<0.001	2.02 (1.98, 2.07)	<0.001
Race		<0.001		<0.001
White	Reference		Reference	
Black	1.02 (0.99, 1.04)	0.221	1.02 (0.99, 1.05)	0.303
AI/AN	1.12 (1.02, 1.22)	0.014	1.19 (1.09, 1.31)	<0.001
AP	0.85 (0.83, 0.87)	<0.001	0.86 (0.83, 0.89)	<0.001
Unknown	0.25 (0.20, 0.32)	<0.001	0.24 (0.18, 0.31)	<0.001
Insurance		<0.001		<0.001
Yes	Reference		Reference	
No	0.89 (0.84, 0.94)	<0.001	0.91 (0.86, 0.96)	<0.001
Unknown	1.08 (1.06, 1.10)	<0.001	1.09 (1.07, 1.11)	<0.001
Marital status		<0.001		<0.001
Married	Reference		Reference	
Unmarried	1.20 (1.17, 1.22)	<0.001	1.15 (1.13, 1.18)	<0.001
Unknown	0.89 (0.85, 0.92)	<0.001	0.87 (0.83, 0.91)	<0.001

Abbreviations: Adeno, Adenocarcinoma; AI/AN, American Indian/Alaska Native; AP, Asian or Pacific Islander; Mucinous, Mucinous cell adenocarcinoma; Signet ring cell, signet ring cell carcinoma.

**TABLE 4:** Multivariable Cox regression analysis of overall and gastric cancer‐specific survival in patients stratified by prior cancer site

Characteristics	Overall adjusted HR	*p*	Gastric cancer‐specific adjusted HR	*p*
Prior cancer site		<0.001		0.061
None	Reference		Reference	
Prostate	1.23 (1.16, 1.29)	<0.001	0.80 (0.75, 0.86)	<0.001
Uterine corpus	1.52 (1.26, 1.83)	<0.001	1.15 (0.91, 1.46)	0.237
Ovary	1.03 (0.75, 1.43)	0.857	0.61 (0.39, 0.96)	0.034
Lung and bronchus	1.61 (1.42, 1.81)	<0.001	0.81 (0.67, 0.97)	0.025
Colon and rectum	1.33 (1.22, 1.45)	<0.001	0.84 (0.38, 1.17)	0.004
Pancreas	1.36 (0.95, 1.94)	0.090	0.67 (0.61, 1.90)	0.159
Liver and intrahepatic bile duct	0.88 (0.60, 1.31)	0.530	0.41 (0.22, 0.77)	0.005
Esophagus	1.48 (1.20, 1.82)	<0.001	0.36 (0.23, 0.59)	<0.001
Urinary bladder	1.32 (1.20, 1.47)	<0.001	0.78 (0.67, 0.91)	0.001
Kidney and renal pelvis	1.11 (0.94, 1.31)	0.234	0.61 (0.47, 0.78)	<0.001
Melanoma of the skin	1.13 (0.96, 1.32)	0.151	0.80 (0.64, 0.99)	0.037
Thyroid	1.06 (0.85, 1.33)	0.613	0.88 (0.67, 1.16)	0.377
Leukemia	1.55 (1.23, 1.96)	<0.001	0.91 (0.65, 1.28)	0.585
Brain and other nervous system	1.20 (1.06, 1.36)	0.005	0.74 (0.62, 0.89)	0.001
Oral cavity and pharynx	1.33 (1.15, 1.53)	<0.001	0.73 (0.59, 0.91)	0.004
Breast	1.23 (1.14, 1.33)	<0.001	0.86 (0.78, 0.96)	0.005
Others	1.31 (1.19, 1.44)	<0.001	0.91 (0.80, 1.03)	0.130

In addition, we did a subgroup analysis and there is no difference in the overall and gastric cancer‐specific survival in patients with gastric cancer between patients with a prior malignancy diagnosed 5 years ago and within 5 years (Table 3, S1 and S3). When stratified by prior cancer site, we found that the overall survival in patients with prior cancer diagnosed 5 years ago was different from patients diagnosed within 5 years including uterine corpus, melanoma of the skin, kidney and renal pelvis, brain and other nervous system (Table S2 and S4). As for the gastric cancer‐specific survival, prior cancer including ovarian, lung and bronchus, colon and rectum, liver and intrahepatic bile duct, esophagus, and urinary bladder were different.

## 4 DISCUSSION

The burgeoning number of cancer survivors has led to an increasing trend of developing a second cancer, which has posed challenges for clinical decisions and oncological practices.^13^ Few studies have focused on survivors since they were commonly excluded from cohort enrollment in clinical trials.^14^ Similar trends were also found in gastric cancer trials,^6^, ^9^ in which individuals with a history of malignancy were excluded from the eligible population. However, there were limited guidelines regarding this exclusion criterion, and it has not been assessed or confirmed based on any sufficient data. Thus, the rationality of the criterion needs to be evaluated on the basis of a large and authoritative population.

Our study sought to investigate the clinical characteristics and prognostic effects of a previous malignancy in patients with gastric cancer. Gastric cancer is increasingly emerging as a second malignancy, similar to other cancer types.^15^, ^16^, ^17^ In the current study, 9.3% of patients with gastric cancer had a prior cancer among more than 70,000 patients, suggesting that prior malignancy‐associated exclusion criteria might restrict a substantial proportion of patients from trial recruitment. Importantly, patients with a prior cancer had smaller tumor sizes, fewer malignant histological types, and less advanced stages and grades, which were attributed to active surveillance in cancer survivors.^18^ In addition, most of the previous cancers were at localized or regional stages. These results might be explained by the fact that those survivors tended to do well and live long enough to develop a second gastric cancer.

It was hypothesized that exposure to prior therapies might reduce treatment tolerance in clinical trials.^17^ The existence of a previous malignancy might interfere with the experimental conduct or the results of the trials.^14^ Another potential reason for excluding patients with prior cancer from clinical trials was that prior malignancies might affect the survival outcomes.^19^ In the overall population, we found that pre‐existing cancer was associated with worse overall survival and better cancer‐specific survival. One possible explanation was that those patients underwent fewer therapies, such as surgery, radiation, and chemotherapy, which were negatively associated with patient prognosis. In addition, the current treatment tolerance of patients is poor because of various reasons such as poor organ function or any decreased reserve capacity after previous treatment for the prior malignancy. Notably, a 5‐year previous malignancy‐related exclusion window has been commonly adopted in clinical trials.^12^ Therefore, we conducted a subgroup survival analysis between patients with a prior cancer diagnosed within 5 years and those without a prior cancer. Similarly, a pre‐existing malignancy increased the overall and decreased the cancer‐specific mortality rates. In addition, there is no difference in the overall and gastric cancer‐specific survival in patients with gastric cancer between patients with a prior malignancy diagnosed 5 years ago and within 5 years. Consistent with our results, researchers have observed an inferior prognosis in patients who had pre‐existing malignancies including lung cancer, prostate cancer, lymphoma, and colon and rectum cancer.^20^, ^21^, ^22^, ^23^, ^24^, ^25^ In contrast, favorable prognostic effects of previous malignancy were found in patients with gastrointestinal malignancies.^25^, ^26^ These conflicting findings regarding the effects of a prior malignancy brought difficulties in determining eligible enrollment in clinical trials.

Among patients who had prior malignancies, prostate, breast, and colon and rectum cancer were the most common types in our study. Similar results were also found in individuals with pancreatic adenocarcinoma, hepatocellular cancer, and lung cancer.^14^, ^19^ This might be partly explained by the fact that prostate cancer and breast cancer have a high incidence in male and female populations, respectively.^5^ Additionally, the favorable prognoses of prostate cancer and breast cancer are attributed to their inert characteristics as well as advanced clinical strategies.^27^ It seemed unlikely that these cancers had adverse effects on patient prognosis due to their indolent process. Interestingly, patients with prostate cancer and breast cancer had worse overall survival in the current study. Similar to gastric cancer, other tumor types, such as urinary bladder, and colon and rectum cancer, are associated with genetic factors and environmental factors, such as diet and smoking.^17^ Therefore, prior urinary bladder, colon and rectum, and gastric cancer may synergistically increase the mortality risk of patients. In addition, pre‐existing lung and bronchus cancer was also associated with inferior survival. This trend might reflect a selection effect that lung cancer itself had a poor survival and prognosis.^28^

Notably, some prior cancer types exerted an opposite effect on the prognostic outcomes of gastric patients. Pre‐existing thyroid cancer did not contribute to worse survival in our study. This result might be attributed to its indolent progression, more intensive medical support, and reduced exposure to risk factors.^17^, ^19^ Similar favorable prognostic effects were observed in ovarian cancer, pancreatic cancer, kidney and renal pelvis cancer, and melanoma of the skin. Multiple gastric cancer trials excluded patients with a history of cancer from the eligible cohort. In contrast, some trials did not exclude or mention this group of patients.^7^, ^9^, ^10^, ^29^, ^30^ We found that prior cancer exerted completely different impacts on the survival prognosis depending on the different prior cancer types. Therefore, excluding patients who had a pre‐existing malignancy in trials should be reconsidered and should be based on the types of previous cancer. Furthermore, the eligibility criteria should be more specific and precise according to the treatment intolerance, patients’ organ function, and health status.^17^ For instance, the certain treatment of a prior cancer, such as radiation, chemotherapy or biological therapy, or the recurrences, might be considered for exclusion rather than the prior cancer diagnosis.

In addition, there were several potential limitations in the present study. First, detailed information on treatments and recurrence was not available in the SEER database. Second, factors of lifestyles, such as alcohol consumption, smoking, and diet, were also inaccessible, which may exert an impact on the prognostic outcomes. Third, specific information regarding the characteristics of the previous malignancy cannot be obtained in this dataset, so we focused on stage and timing only. Prior malignancies were grouped into 16 types based on commonly used categories; therefore, less common cancer types were not evaluated in our study due to the limited number of patients. Last, there might be selective and surveillance biases due to the retrospective nature of the study.

In conclusion, the clinical characteristics were significantly different between patients with and without previous malignancies. Prior malignancies had an adverse impact on the overall survival of patients with gastric cancer, and this adverse effect was more obvious in patients with initial cancer types including prostate, uterine corpus, lung and bronchus, colon and rectum, esophagus, urinary bladder, leukemia, brain and other nervous system, oral cavity and pharynx and breast cancer. Additionally, prior cancers, such as ovarian cancer, pancreatic cancer, kidney and renal pelvis cancer, melanoma of the skin, and thyroid cancer, were not associated with worse outcomes. Therefore, the exclusion and inclusion of patients who had previous malignancies should be reconsidered according to the specific malignancy types.

## CONFLICTS OF INTEREST

The authors declare no conflict of interest.

## Supporting information

Figure S1

Figure S2

Tables S1‐S4

## Data Availability

All data were obtained from SEER database. https://seer.cancer.gov/data/
