# Supplementary figures and images for: The impact of a prior malignancy on outcomes in gastric cancer patients
Source: Cancer Med. 2021 Jan 19;10(4):1457–70. doi: 10.1002/cam4.3722 (PMC7926016; doi:10.1002/cam4.3722)

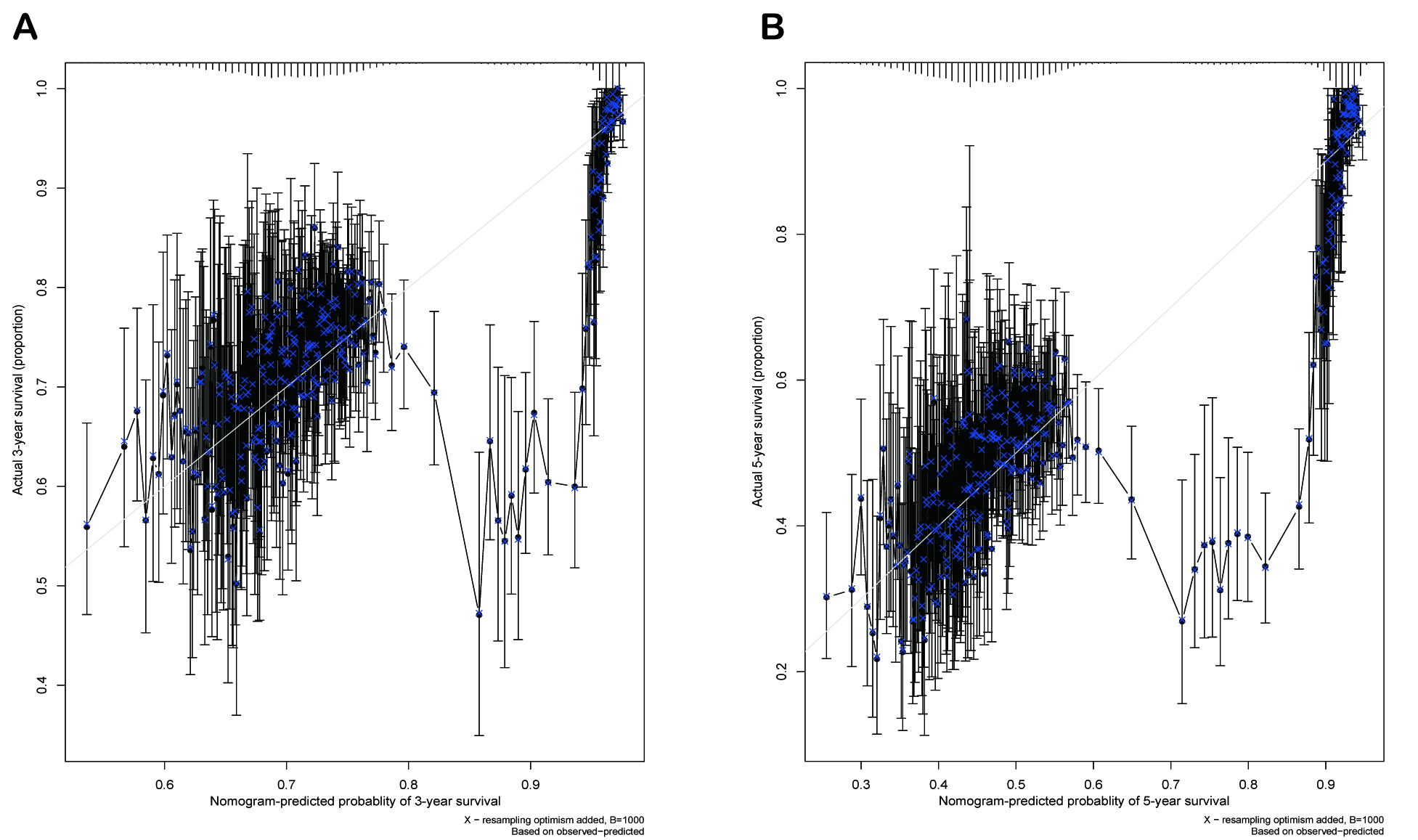

Supplement: Supplementary file 1 — Figure S1 [file CAM4-10-1457-s001.tif]

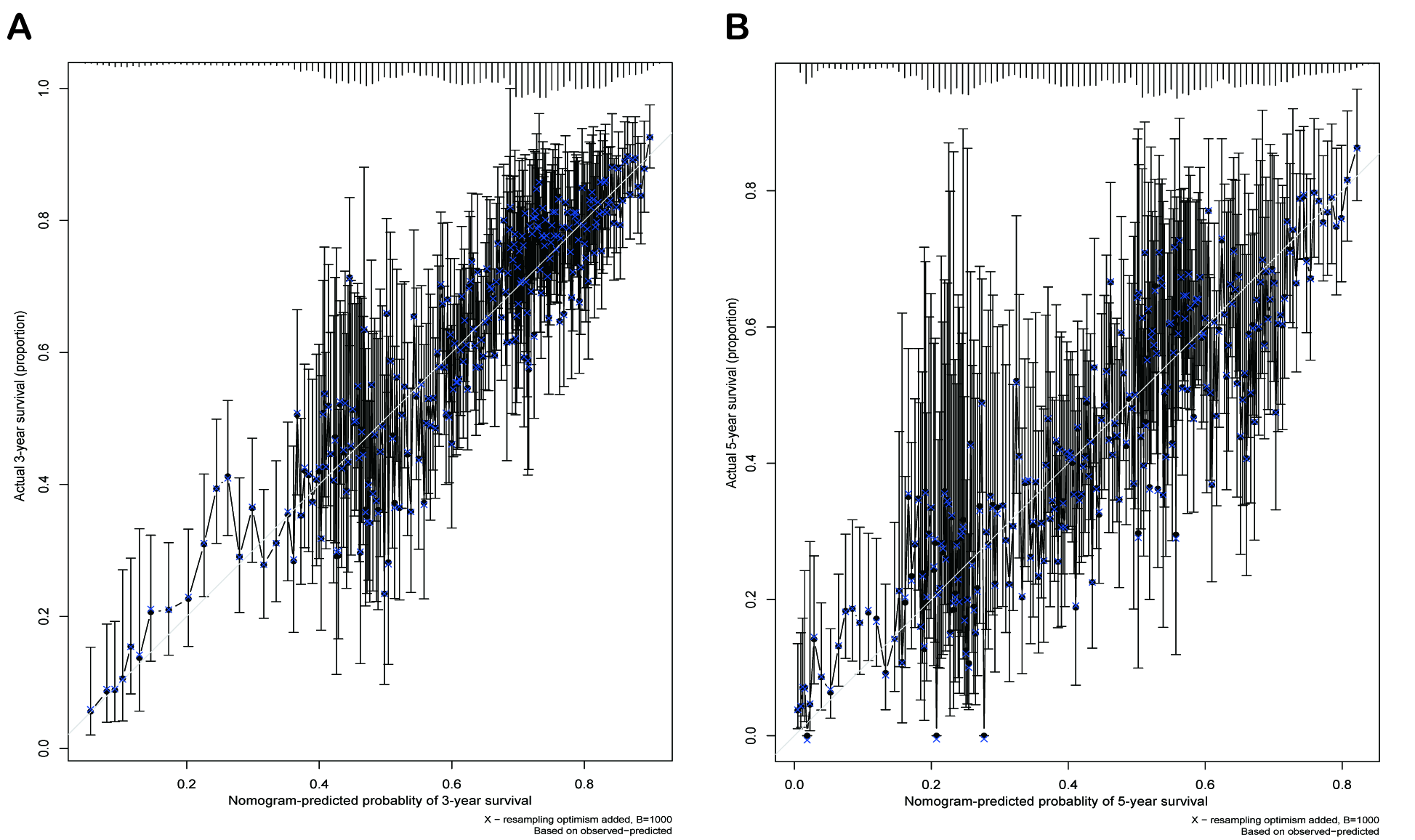

Supplement: Supplementary file 2 — Figure S2 [file CAM4-10-1457-s002.tif]
