# Supplementary material for: The impact of a prior malignancy on outcomes in gastric cancer patients
Source: Cancer Med. 2021 Jan 19;10(4):1457–70. doi: 10.1002/cam4.3722 (PMC7926016; doi:10.1002/cam4.3722)
Supplement: Supplementary file 3 — Tables S1‐S4 [file CAM4-10-1457-s003.docx]

**Table S1. Multivariable Cox regression analysis of overall and gastric cancer-specific survival in patients with gastric cancer (prior cancer diagnosed ≤5 years).**

| **Characteristics** | **Overall adjusted HR** | **P** | **Gastric cancer-specific adjusted HR** | **P** |
| --- | --- | --- | --- | --- |
| **Prior cancer** |  |  |  |  |
| Yes | Reference |  | Reference |  |
| No | 0.78 (0.74, 0.83) | <0.001 | 1.28 (1.19, 1.37) | <0.001 |
| **Age at diagnoses** |  |  |  |  |
| ≤ 70 years | Reference |  | Reference |  |
| > 70 years | 1.26 (1.16, 1.37) | <0.001 | 1.42 (1.25, 1.60) | <0.001 |
| **Sex** |  | <0.001 |  | <0.001 |
| Male | Reference |  | Reference |  |
| Female | 0.89 (0.87, 0.91) | <0.001 | 0.90 (0.88, 0.92) | <0.001 |
| **Tumour Site** |  | <0.001 |  | <0.001 |
| Cardia | Reference |  | Reference |  |
| Body/fundus | 0.91 (0.88, 0.94) | <0.001 | 0.93 (0.90, 0.96) | <0.001 |
| Antrum/pylorus | 1.00 (0.97, 1.02) | 0.772 | 1.01 (0.98, 1.04) | 0.493 |
| Others | 1.01 (1.00, 1.04) | 0.267 | 1.04 (1.02, 1.07) | 0.002 |
| **Histological Type** |  | <0.001 |  | <0.001 |
| Adeno | Reference |  | Reference |  |
| Mucinous | 1.10 (1.03, 1.17) | 0.003 | 1.14 (1.06, 1.22) | <0.001 |
| Signet ring cell | 1.11 (1.08, 1.14) | <0.001 | 1.14 (1.11, 1.17) | <0.001 |
| Others | 0.83 (0.80, 0.87) | 0.834 | 0.83 (0.79, 0.86) | <0.001 |
| **Tumour Size** |  | <0.001 |  | <0.001 |
| ≤ 1 cm | Reference |  | Reference |  |
| 1-3 cm | 1.55 (1.46, 1.65) | <0.001 | 1.94 (1.79, 2.11) | <0.001 |
| 3-5 cm | 1.95 (1.83, 2.07) | <0.001 | 2.51 (2.31, 2.72) | <0.001 |
| >5 cm | 2.09 (1.97, 2.21) | <0.001 | 2.76 (2.54, 2.99) | <0.001 |
| Unknown | 2.07 (1.95, 2.19) | <0.001 | 2.69 (2.49, 2.92) | <0.001 |
| **AJCC stage (6th)** |  | <0.001 |  | <0.001 |
| I | Reference |  | Reference |  |
| II | 1.62 (1.56, 1.68) | <0.001 | 1.82 (1.75, 1.90) | <0.001 |
| III | 2.18 (2.10, 2.26) | <0.001 | 2.50 (2.40, 2.61) | <0.001 |
| IV | 2.83 (2.75, 2.92) | <0.001 | 3.10 (3.09, 3.30) | <0.001 |
| Others | 1.00 (0.96, 1.03) | 0.808 | 1.05 (1.01, 1.09) | 0.022 |
| **Grade** |  | <0.001 |  | <0.001 |
| Well | Reference |  | Reference |  |
| Moderately | 1.79 (1.69, 1.88) | <0.001 | 1.93 (1.85, 2.02) | <0.001 |
| Poorly | 2.21 (2.09, 2.33) | <0.001 | 2.71 (2.59, 2.83) | <0.001 |
| Undifferentiated | 2.23 (2.06, 2.41) | <0.001 | 3.62 (3.50, 3.75) | <0.001 |
| Unknown | 1.51 (1.43, 1.59) | <0.001 | 1.08 (1.04, 1.13) | <0.001 |
| **Surgery** |  | <0.001 |  | <0.001 |
| Yes | Reference |  | Reference |  |
| No | 3.25 (3.17, 3.34) | <0.001 | 3.45 (3.35, 3.56) | <0.001 |
| Unknown | 2.22 (2.06, 2.40) | <0.001 | 2.49 (2.28, 2.71) | <0.001 |
| **Radiation** |  | <0.001 |  | <0.001 |
| Yes | Reference |  | Reference |  |
| No | 0.92 (0.89, 0.95) | <0.001 | 0.89 (0.86, 0.92) | <0.001 |
| Unknown | 1.04 (0.70, 1.53) | 0.862 | 1.11 (0.73,1.69) | 0.614 |
| **Chemotherapy** |  | <0.001 |  | <0.001 |
| Yes | Reference |  | Reference |  |
| No/Unknown | 2.07 (2.02, 2.11) | <0.001 | 2.03 (1.98, 2.08) | <0.001 |
| **Race** |  | <0.001 |  | <0.001 |
| White | Reference |  | Reference |  |
| Black | 1.01 (0.99, 1.04) | 0.337 | 1.01 (0.98, 1.04) | 0.435 |
| AI/AN | 1.13 (1.04, 1.24) | 0.006 | 1.21 (1.10, 1.33) | <0.001 |
| AP | 0.85 (0.82, 0.87) | <0.001 | 0.85 (0.83, 0.88) | <0.001 |
| Unknown | 0.25 (0.20, 0.32) | <0.001 | 0.24 (0.18, 0.31) | <0.001 |
| **Insurance** |  | <0.001 |  | <0.001 |
| Yes | Reference |  | Reference |  |
| No | 0.89 (0.84, 0.94) | <0.001 | 0.91 (0.85, 0.96) | 0.001 |
| Unknown | 1.08 (1.06, 1.11) | <0.001 | 1.10 (1.07, 1.12) | <0.001 |
| **Marital status** |  | <0.001 |  | <0.001 |
| Married | Reference |  | Reference |  |
| Unmarried | 1.20 (1.18, 1.22) | <0.001 | 1.15 (1.13, 1.18) | <0.001 |
| Unknown | 0.88 (0.85, 0.92) | <0.001 | 0.87 (0.83, 0.91) | <0.001 |

Abbreviation: AI/AN, American Indian/Alaska Native; AP, Asian or Pacific Islander; Adeno, Adenocarcinoma; Mucinous, Mucinous cell adenocarcinoma; Signet ring cell, signet ring cell carcinoma.

**Table S2. Multivariable Cox regression analysis of overall and gastric cancer-specific survival in patients stratified by prior cancer site (prior cancer diagnosed ≤5 years).**

| **Characteristics** | **Overall adjusted HR** | **P** | **Gastric cancer-specific**  **adjusted HR** | **P** |
| --- | --- | --- | --- | --- |
| **Prior cancer site** |  | <0.001 |  | 0.061 |
| **None** | Reference |  | Reference |  |
| **Prostate** | 1.23 (1.13, 1.33) | <0.001 | 0.87 (0.78, 0.97) | 0.009 |
| **Uterine corpus** | 1.34 (0.97, 1.85) | 0.077 | 1.07 (0.71, 1.60) | 0.747 |
| **Ovary** | 0.81 (0.52, 1.27) | 0.359 | 0.34 (0.16, 0.72) | 0.005 |
| **Lung & bronchus** | 1.60 (1.39, 1.85) | <0.001 | 0.76 (0.60, 0.95) | 0.017 |
| **Colon & rectum** | 1.25 (1.09, 1.42) | 0.001 | 0.71 (0.59, 0.87) | 0.001 |
| **Pancreas** | 1.32 (0.85, 2.05) | 0.212 | 0.43 (0.18, 1.03) | 0.057 |
| **Liver & intrahepatic bile duct** | 1.04 (0.66, 1.64) | 0.857 | 0.46 (0.22, 0.96) | 0.039 |
| **Esophagus** | 1.54 (1.22, 1.96) | <0.001 | 0.32 (0.18, 0.59) | <0.001 |
| **Urinary bladder** | 1.40 (1.20, 1.63) | <0.001 | 0.89 (0.72, 1.11) | 0.307 |
| **Kidney & renal pelvis** | 0.97 (0.76, 1.23) | 0.787 | 0.58 (0.41, 0.82) | 0.002 |
| **Melanoma of the skin** | 1.32 (1.04, 1.67) | 0.025 | 0.84 (0.60, 1.18) | 0.304 |
| **Thyroid** | 0.83 (0.55, 1.26) | 0.381 | 0.64 (0.38, 1.08) | 0.092 |
| **Leukemia** | 1.63 (1.19, 2.22) | 0.002 | 1.09 (0.71, 1.68) | 0.698 |
| **Brain & other nervous system** | 1.08 (0.90, 1.31) | 0.414 | 0.62(0.46, 0.83) | 0.001 |
| **Oral cavity & pharynx** | 1.27 (1.03, 1.56) | 0.027 | 0.69 (0.50, 0.95) | 0.024 |
| **Breast** | 1.18 (1.05, 1.34) | 0.008 | 0.75 (0.62, 0.89) | 0.005 |
| **Others** | 1.51 (1.31, 1.74) | <0.001 | 0.93 (0.76, 1.14) | 0.496 |

**Table S3. Multivariable Cox regression analysis of overall and gastric cancer-specific survival in patients with gastric cancer (prior cancer diagnosed >5 years).**

| **Characteristics** | **Overall adjusted HR** | **P** | **Gastric cancer-specific adjusted HR** | **P** |
| --- | --- | --- | --- | --- |
| **Prior cancer** |  |  |  |  |
| Yes | Reference |  | Reference |  |
| No | 0.79 (0.76, 0.83) | <0.001 | 1.21 (1.14, 1.29) | <0.001 |
| **Age at diagnoses** |  |  |  |  |
| ≤ 70 years | Reference |  | Reference |  |
| > 70 years | 1.41 (1.30, 1.53) | <0.001 | 1.41 (1.26, 1.58) | <0.001 |
| **Sex** |  | <0.001 |  | <0.001 |
| Male | Reference |  | Reference |  |
| Female | 0.89 (0.88, 0.91) | <0.001 | 0.91 (0.89, 0.93) | <0.001 |
| **Tumour Site** |  | <0.001 |  | <0.001 |
| Cardia | Reference |  | Reference |  |
| Body/fundus | 0.90 (0.88, 0.93) | <0.001 | 0.93 (0.90, 0.96) | <0.001 |
| Antrum/pylorus | 1.00 (0.97, 1.02) | 0.807 | 1.02 (0.99, 1.06) | 0.119 |
| Others | 1.01 (0.99, 1.03) | 0.422 | 1.05 (1.02, 1.08) | <0.001 |
| **Histological Type** |  | <0.001 |  | <0.001 |
| Adeno | Reference |  | Reference |  |
| Mucinous | 1.11 (1.04, 1.18) | 0.002 | 1.13 (1.06, 1.21) | <0.001 |
| Signet ring cell | 1.11 (1.09, 1.14) | <0.001 | 1.15 (1.12, 1.18) | <0.001 |
| Others | 0.84 (0.81, 0.87) | <0.001 | 0.83 (0.80, 0.86) | <0.001 |
| **Tumour Size** |  | <0.001 |  | <0.001 |
| ≤ 1 cm | Reference |  | Reference |  |
| 1-3 cm | 1.56 (1.47, 1.66) | <0.001 | 1.96 (1.80, 2.13) | <0.001 |
| 3-5 cm | 1.97 (1.86, 2.10) | <0.001 | 2.54 (2.34, 2.76) | <0.001 |
| >5 cm | 2.10 (1.98, 2.23) | <0.001 | 2.79 (2.57, 3.02) | <0.001 |
| Unknown | 2.09 (1.97, 2.21) | <0.001 | 2.73 (2.52, 2.95) | <0.001 |
| **AJCC stage (6th)** |  | <0.001 |  | <0.001 |
| I | Reference |  | Reference |  |
| II | 1.62 (1.56, 1.68) | <0.001 | 1.92 (1.84, 2.01) | <0.001 |
| III | 2.17 (2.09, 2.25) | <0.001 | 2.71 (2.60, 2.83) | <0.001 |
| IV | 2.83 (2.75, 2.91) | <0.001 | 3.60 (3.48, 3.72) | <0.001 |
| Others | 1.00 (0.96, 1.03) | 0.756 | 1.08 (1.04, 1.13) | <0.001 |
| **Grade** |  | <0.001 |  | <0.001 |
| Well | Reference |  | Reference |  |
| Moderately | 1.79 (1.70, 1.89) | <0.001 | 2.08 (1.94, 2.23) | <0.001 |
| Poorly | 2.20 (2.09, 2.32) | <0.001 | 2.71 (2.53, 2.90) | <0.001 |
| Undifferentiated | 2.21 (2.04, 2.39) | <0.001 | 2.77 (2.52, 3.03) | <0.001 |
| Unknown | 1.51 (1.43, 1.60) | <0.001 | 1.80 (1.68, 1.93) | <0.001 |
| **Surgery** |  | <0.001 |  | <0.001 |
| Yes | Reference |  | Reference |  |
| No | 3.23 (3.14, 3.31) | <0.001 | 3.46 (3.36, 3.57) | <0.001 |
| Unknown | 2.24 (2.07, 2.42) | <0.001 | 2.52 (2.31, 2.75) | <0.001 |
| **Radiation** |  | <0.001 |  | <0.001 |
| Yes | Reference |  | Reference |  |
| No | 0.92 (0.89, 0.95) | <0.001 | 0.89 (0.85, 0.92) | <0.001 |
| Unknown | 1.06 (0.72, 1.56) | 0.781 | 1.12 (0.74,1.69) | 0.605 |
| **Chemotherapy** |  | <0.001 |  | <0.001 |
| Yes | Reference |  | Reference |  |
| No/Unknown | 2.07 (2.03, 2.12) | <0.001 | 2.03 (1.99, 2.08) | <0.001 |
| **Race** |  | <0.001 |  | <0.001 |
| White | Reference |  | Reference |  |
| Black | 1.01 (0.99, 1.04) | 0.310 | 1.01 (0.98, 1.04) | 0.435 |
| AI/AN | 1.11 (1.02, 1.21) | 0.021 | 1.19 (1.08, 1.31) | <0.001 |
| AP | 0.85 (0.83, 0.87) | <0.001 | 0.86 (0.83, 0.88) | <0.001 |
| Unknown | 0.25 (0.20, 0.32) | <0.001 | 0.23 (0.18, 0.31) | <0.001 |
| **Insurance** |  | <0.001 |  | <0.001 |
| Yes | Reference |  | Reference |  |
| No | 0.89 (0.84, 0.94) | <0.001 | 0.91 (0.86, 0.97) | 0.002 |
| Unknown | 1.08 (1.06, 1.11) | <0.001 | 1.09 (1.07, 1.11) | <0.001 |
| **Marital status** |  | <0.001 |  | <0.001 |
| Married | Reference |  | Reference |  |
| Unmarried | 1.20 (1.17, 1.22) | <0.001 | 1.15 (1.13, 1.18) | <0.001 |
| Unknown | 0.89 (0.85, 0.93) | <0.001 | 0.88 (0.84, 0.92) | <0.001 |

Abbreviation: AI/AN, American Indian/Alaska Native; AP, Asian or Pacific Islander; Adeno, Adenocarcinoma; Mucinous, Mucinous cell adenocarcinoma; Signet ring cell, signet ring cell carcinoma.

**Table S4. Multivariable Cox regression analysis of overall and gastric cancer-specific survival in patients stratified by prior cancer site (prior cancer diagnosed >5 years).**

| **Characteristics** | **Overall adjusted HR** | **P** | **Gastric cancer-specific**  **adjusted HR** | **P** |
| --- | --- | --- | --- | --- |
| **Prior cancer site** |  | <0.001 |  | 0.061 |
| **None** | Reference |  | Reference |  |
| **Prostate** | 1.22 (1.15, 1.31) | <0.001 | 0.76 (0.69, 0.83) | <0.001 |
| **Uterine corpus** | 1.63 (1.30, 2.04) | <0.001 | 1.21 (0.90, 1.62) | 0.206 |
| **Ovary** | 1.41 (0.89, 2.26) | 0.146 | 1.16 (0.65, 2.05) | 0.614 |
| **Lung & bronchus** | 1.57 (1.26, 1.96) | <0.001 | 0.92 (0.67, 1.26) | 0.606 |
| **Colon & rectum** | 1.40 (1.25, 1.56) | <0.001 | 0.93 (0.80, 1.09) | 0.370 |
| **Pancreas** | 1.46 (0.80, 2.64) | 0.217 | 1.14 (0.54, 2.42) | 0.725 |
| **Liver & intrahepatic bile duct** | 0.58 (0.26, 1.30) | 0.187 | 0.34 (0.11, 1.05) | 0.060 |
| **Esophagus** | 1.21 (0.78, 1.88) | 0.391 | 0.46 (0.21, 1.02) | 0.057 |
| **Urinary bladder** | 1.26 (1.09, 1.46) | 0.002 | 0.69 (0.56, 0.85) | 0.001 |
| **Kidney & renal pelvis** | 1.27 (1.01, 1.59) | 0.043 | 0.64 (0.45, 0.92) | 0.015 |
| **Melanoma of the skin** | 1.01 (0.81, 1.26) | 0.923 | 0.77 (0.59, 1.02) | 0.065 |
| **Thyroid** | 1.22 (0.93, 1.60) | 0.153 | 1.05 (0.76, 1.45) | 0.775 |
| **Leukemia** | 1.45 (1.02, 2.07) | 0.038 | 0.70 (0.40, 1.24) | 0.222 |
| **Brain & other nervous system** | 1.31 (1.11, 1.55) | 0.002 | 0.86 (0.68, 1.08) | 0.182 |
| **Oral cavity & pharynx** | 1.37 (1.12, 1.67) | 0.002 | 0.76 (0.57, 1.02) | 0.065 |
| **Breast** | 1.26 (1.14, 1.39) | <0.001 | 0.94 (0.78, 0.96) | 0.358 |
| **Others** | 1.20 (1.05, 1.36) | 0.006 | 0.90 (0.77, 1.06) | 0.205 |
